# The impact of working conditions on the mental health of workers in a confectionery factory

**DOI:** 10.1192/j.eurpsy.2024.1241

**Published:** 2024-08-27

**Authors:** H. Daoud, I. Sellami, C. Ben Chabene, M. A. Ghrab, A. Haddar, M. Hajjaji, K. Jmal Hammami, M. L. Masmoudi

**Affiliations:** ^1^Occupational Medicine, Hedi Chaker Hospital; ^2^Sfax University, Sfax; ^3^Occupational Medicine, Tunis, Tunisia

## Abstract

**Introduction:**

Mental health is a critical factor influencing employee well-being and performance in companies. However, many factors within professional environments can either positively or negatively impact employees’ psychological well-being.

**Objectives:**

This study aims to assess mental health among workers in a confectionery factory and its association with job satisfaction.

**Methods:**

We conducted a cross-sectional study among workers in a private confectionery in Sfax. Questionnaires and workplace assessments were collected over a period from December 2022 to July 2023 using a pre-established questionnaire. Mental health assessment was performed using the 21-item Depression, Anxiety, and Stress Questionnaire (DASS21). The degree of job satisfaction was assessed using a visual analog scale ranging from 0 to 10.

**Results:**

Our study included 200 participants, with 61% being female. Severe to very severe symptoms of depression, anxiety, and stress were found in 4.5%, 17%, and 10.5% of our participants, respectively. Among our workers, 22.5% reported being not very satisfied or not satisfied. Bivariate analysis revealed lower levels of satisfaction among the most anxious (p = 0.000), the most depressed (p = 0.000), and the most stressed (p = 0.000) workers.

**Conclusions:**

The decline in mental health is closely linked to job dissatisfaction. Implementing measures to enhance employee job satisfaction and providing adequate support resources for mental well-being are essential steps to promote a healthier workplace and improve employee well-being.

**Disclosure of Interest:**

None Declared

